# Commensal Neisseria Inhibit Porphyromonas Gingivalis Invasion of Gingival Epithelial Cells

**DOI:** 10.3290/j.ohpd.b5866430

**Published:** 2024-12-02

**Authors:** Shota Fukuda, Tomoki Akatsu, Akihiko Fujii, Sawako Kawano, Yoshihiko Minegishi, Noriyasu Ota

**Affiliations:** a Shota Fukuda Research Scientist, Biological Science Research, Kao Corporation. Experimental design, performed experiments, analysed the data, wrote the manuscript, and contributed substantially to the discussion.; b Tomoki Akatsu Research Scientist, Biological Science Research, Kao Corporation. Provided advice on the experimental design, reviewed the manuscript, and contributed substantially to the discussion.; c Akihiko Fujii Research Scientist, Biological Science Research, Kao Corporation. Provided advice on the experimental design, reviewed the manuscript, and contributed substantially to the discussion.; d Sawako Kawano Research Scientist, Biological Science Research, Kao Corporation. Performed experiments, reviewed the manuscript, and contributed substantially to the discussion.; e Yoshihiko Minegishi Manager, Biological Science Research, Kao Corporation. Contributed substantially to the discussion, edited the manuscript, and approved the final manuscript.; f Noriyasu Ota Director, Biological Science Research, Kao Corporation. Advisor, read and approved the final manuscript.

**Keywords:** Neisseria, oral probiotics, invasion inhibition, periodontitis

## Abstract

**Purpose:**

Periodontal disease is caused by periodontal invasion by pathogens such as *Porphyromonas gingivalis*. Although recent metagenomic analyses have shown that oral commensal bacteria are abundant in the mouth of healthy individuals, few studies have experimentally verified the benefits and functions of oral commensal bacteria in periodontal diseases. In this study, we focused on *Neisseria* among the oral commensal bacteria and aimed to experimentally verify its effects on *P. gingivalis* invasion.

**Materials and Methods:**

We evaluated the inhibitory effect of *Neisseria* spp. on *P. gingivalis* invasion using a flow cytometry-based invasion assay and analysed bacterial interactions by visualisation using scanning electron microscopy. Furthermore, we constructed a new experimental pre-mixed culture system that reproduced the interaction environment to evaluate the relevance of this interaction in invasion inhibition.

**Results:**

Flow cytometry-based invasion assays showed that all *Neisseria* spp. inhibited *P. gingivalis* invasion, with *Neisseria mucosa* and *Neisseria elongata* being particularly effective. Interaction analysis using scanning electron microscopy showed that *N. mucosa* and *N. elongata*, which have strong inhibitory effects on *P. gingivalis* invasion, interacted with *P. gingivalis* at high frequencies.

**Conclusion:**

Commensal *Neisseria* was found to exert a beneficial function by directly interacting with *P. gingivalis* and inhibiting its invasion of gingival epithelial cells. These results suggest that *Neisseria*, as a probiotic or synbiotic oral commensal, may represent an innovative approach to preventing periodontal disease.

Periodontal disease is caused by bacterial infection,^7^ which leads to periodontal tissue destruction through alveolar bone resorption and inflammation, resulting in tooth loss.^5,31^ Porphyromonas gingivalis — a gram-negative, anaerobic, black-pigmented bacterium — is known to be a representative periodontal pathogen and a component of the ‘red complex’ with *Treponema denticola* and *Tannerella forsythia*.^18^ *P. gingivalis* has been reported to infect gingival epithelial cells,^52^ resulting in epithelial cell dysfunction and periodontal tissue destruction. Also, virulent factors of *P. gingivalis*, such as the capsule, outer membrane proteins, lipopolysaccharides, proteases such as gingipains, collagenases, haemolysin, trypsin proteases, hemagglutinins, and fimbriae, are involved in colonisation and invasion.^14,25,34,53,54^ During an infection, *P. gingivalis* can penetrate deep into epithelial cells, eventually invading tissues^1^ and circulating throughout the body via the red blood cells.^6^ Systemically circulating *P. gingivalis* has been reported to invade the brain and contribute to the progression of Alzheimer’s disease,^36^ invade human colonic artery endothelial cells, contribute to atherosclerosis,^37^ infect immortalised human oral epithelial cells, and induce oral squamous cell carcinoma.^15^ Therefore, *P. gingivalis* infection is not only a serious factor in the progression of periodontal disease but can also cause systemic diseases.

Over 700 bacterial species inhabit the oral cavity, including both periodontal pathogens, such as *P. gingivalis*, and non-pathogenic commensal bacteria.^2,44^ Recent metagenomic analyses have shown that Neisseria, a gram-negative bacteria, is more abundant in healthy individuals than in individuals with periodontal disease.^8,21,49,50^ More than 20 *Neisseria* spp. exist^29^ including *Neisseria mucosa*, *Neisseria sicca*, and *Neisseria elongata* in plaques, and *Neisseria flavescens*, *Neisseria flava*, and *Neisseria subflava* in saliva.^13^ These reports indicate that oral commensal *Neisseria* may be beneficial in maintaining oral health. However, no studies have experimentally verified the effect of *Neisseria* on periodontal disease and periodontal pathogens, and it is unclear whether *Neisseria* plays a beneficial role against periodontal disease. Therefore, we investigated the effects of *Neisseria* spp. on *P. gingivalis* invasion using *in-vitro* evaluation systems and microscopic visualisation techniques.

## MATERIALS AND METHODS

### Cells and Culture Conditions

The human gingival epithelial cell line, Ca9-22 (JCRB0625), is an established transformed human gingival cell line that has been used in previous studies^43^ as a culture model of oral epithelial cells; the cell line was obtained from the Japanese Collection of Research Bioresources (Tokyo, Japan). Ca9-22 cells were cultured in Dulbecco’s modified Eagle medium (DMEM) (Gibco^TM^ Life Technologies, Carlsbad, CA, USA) supplemented with 10% foetal bovine serum (FBS) (Sigma-Aldrich, St Louis, MO, USA) and 1% penicillin-streptomycin (Gibco^TM^ Life Technologies, Carlsbad, CA, USA) at 37°C in 5% CO_2_. The cells were used in this study based on information from previous studies^43^ that tested similar in-vitro evaluation systems.

### Bacterial Strains and Growth Conditions

The periodontal pathogen strains used in this study were *P. gingivalis *strain ATCC 33277 and *Fusobacterium nucleatum *strain ATCC 23726. These bacteria were cultured on a blood agar plate (BD BBL^TM^ anaerobic Columbia RS blood agar plate, BD Biosciences, Franklin Lakes, NJ, USA) for colony formation, and then 5–10 colonies were transferred to Gifu Anaerobic Medium (GAM) broth (Nissui Pharmaceutical, Tokyo, Japan) supplemented with 5.0 µg mL^–1^ Hemin, 17.4 µg mL^–1^ K2HPO4, and 1.0 µg mL^–1^ vitamin K at 37°C under anaerobic conditions. The Neisseria strains used in this study were *N. mucosa* strain JCM 12992, *N. elongata* strain ATCC 25295, *N. sicca* strain ATCC 29256, *N. flava* strain ATCC 14221, *N. flavescens* strain ATCC 13120, and* N. subflava *strain ATCC 49275. These bacteria were cultured on Brain Heart Infusion (BHI) agar (BD Biosciences, Franklin Lakes, NJ, USA), and then 5–10 colonies were transferred to BHI broth (BD Biosciences, Franklin Lakes, NJ, USA) at 37°C in 5% CO2.

*P. gingivalis* and *F. nucleatum* were pre-incubated for 24 h and then inoculated into fresh GAM broth supplemented with 5.0 µg mL^–1^ Hemin, 17.4 µg mL^–1^ K2HPO4, and 1.0 µg mL^–1^ vitamin K for a further 24 h with the addition of 1/1,000 volume of the pre-incubated culture at 37°C under anaerobic conditions. *Neisseria* spp. were pre-incubated for 48 h and then inoculated into fresh BHI broth for a further 48 h with the addition of 1/100 volume of the pre-incubated culture at 37°C in 5% CO2.

### Carboxylfluorescein Diacetate Succinimidyl Ester Labelling of *P. gingivalis*

*P. gingivalis* cultures were centrifuged at 4,500 g for 10 min at 24°C, and the bacteria were collected. The recovered bacteria were washed with phosphate-buffered saline (PBS) (DPBS without calcium and magnesium; Gibco^TM^ Life Technologies, Carlsbad, CA, USA) and serum-free DMEM at 4,500 g for 5 min at 24°C. The bacteria were adjusted to OD600 = 2.0 and incubated in 10 µM carboxylfluorescein diacetate succinimidyl ester (CFSE) (Wako, Osaka, Japan) dissolved in serum-free DMEM at 37°C in the dark for 30 min under anaerobic conditions. CFSE-labelled *P. gingivalis* cells were prepared and washed twice with serum-free DMEM.

### Epithelial Cell Invasion Assay

Ca9-22 cells were seeded in 12-well plates at 0.2×10^6^ cells per well and incubated for 48 h. The medium was removed and substituted with the serum-free DMEM after washing with PBS. The multiplicity of infection (MOI) was calculated based on the number of cells per well when they reached confluence. Then, *Neisseria* spp. (MOI = 100, 200, 500) or *F. nucleatum* (MOI = 100, 200) suspended in serum-free DMEM were added and pre-incubated for 1 h at 37°C in 5% CO2. CFSE-labelled *P. gingivalis* (MOI = 500) was added and incubated for 2 h at 37°C in 5% CO2 under 80 rpm shaking conditions (n = 3).

### Flow Cytometry Analysis

After infecting Ca9-22 cells with CFSE-labelled *P. gingivalis*, unadhered bacteria were removed by washing twice with PBS. External adherent bacteria were then killed by incubation in DMEM containing 300 µg mL^–1^ of gentamicin (Wako, Osaka, Japan) and 200 µg mL^–1^ of metronidazole (Wako, Osaka, Japan) for 1 h. This concentration of antibiotics was sufficient to kill 10^8^ bacteria mL^–1^ in 1 h at 37°C. After antibiotic exposure, the cells were detached using 0.25% Trypsin-EDTA (Gibco^TM^ Life Technologies, Carlsbad, CA, USA) after washing twice with PBS. Then, DMEM (containing 1% FBS) was added, and the cells were collected in 1.5 mL tubes. The collected cells were centrifuged at 400 g for 5 min at 4°C. The supernatant was then removed. Cells were pipetted with 4% paraformaldehyde phosphate buffer (Wako, Osaka, Japan) and fixed for 15 min at 4°C in the dark. After fixation, PBS (containing 2% FBS) was added, the cells were mixed and centrifuged at 600 g for 5 min at 4°C, and the cells were resuspended in PBS (containing 2% FBS) for analysis. Flow cytometry (BD FACSVerse^TM^, BD Biosciences, Franklin Lakes, NJ, USA) was used for analysis.

### Antibacterial Assay

The bacteria were prepared in the same manner as that for the epithelial cell invasion assay without CFSE labelling. The same amounts of *P. gingivalis* and *Neisseria* spp. used in the epithelial cell invasion assay were added to 12-well plates without Ca9-22 cells and incubated for 2 h. After incubation, the culture medium was collected in 1.5 mL tubes and mixed well. The collected culture was diluted, plated on a blood agar plate, and incubated at 37°C under anaerobic conditions for 2 days (under such anaerobic conditions, *Neisseria* spp. do not colonise during this experiment). We determined the number of viable *P. gingivalis* by counting the colonies (n = 3).

### Scanning Electron Microscopy

Semi-confluent Ca9-22 cells on 15-mm-diameter glass coverslips were co-cultured with *P. gingivalis* (MOI = 500) and/or *Neisseria* spp. (MOI = 500) without antibiotics for 30 min. After infecting Ca9-22 cells with *P. gingivalis*, unadhered bacteria were removed by washing with 10 mM HEPES buffer (pH 7.4) (Gibco^TM^ Life Technologies, Carlsbad, CA, USA) and fixed in 2.5% glutaraldehyde (Wako, Osaka, Japan) in 10 mM HEPES buffer for 2 h at 4°C. Following serial dehydration, all samples were coated with gold using a smart coater (DII-29010SCTR; Japan Electron Optics Laboratory, Tokyo, Japan). Ca9-22 cells and bacteria were visualised using scanning electron microscopy (SEM) (JSM-6510; Japan Electron Optics Laboratory, Tokyo, Japan) at an accelerating voltage of 10.0 kV to directly observe bacterial–bacteria and bacterial–host-cells interactions.

### Pre-mixed Culture and Simultaneous Culture Systems

For the pre-mixed culture system, the prepared *Neisseria* spp. (MOI = 500) or *F. nucleatum* (MOI = 200) was mixed with CFSE-labelled *P. gingivalis* (MOI = 500) for 1 h at 37°C under 80 rpm shaking conditions. Then, the mixture was added to Ca9-22 cells and incubated for 2 h at 37°C.

For the simultaneous culture system, the bacteria were prepared according to the procedure used for the epithelial cell invasion assay. *Neisseria* spp. (MOI = 500) or *F. nucleatum* (MOI = 200) and CFSE-labelled *P. gingivalis* (MOI = 500) were added to Ca9-22 cells simultaneously and incubated for 2 h at 37°C without pre-incubation. The same procedure used for the epithelial invasion assay was used for subsequent sample preparation and flow cytometric analysis (*Neisseria* spp.; n = 6, *F. nucleatum*; n = 3).

### Statistical Analysis

The mean fluorescence intensity was calculated from the fluorescence intensity obtained from fluorescein isothiocyanate channels of 500,000 cells gated according to forward scatter and side scatter, and the fluorescence intensity was used to assess *P. gingivalis* invasion. To compare the invasive ability of different strains, the invasion index was calculated as follows: [mean fluorescence intensity (MFI) of infected cells–MFI of negative control cells]/MFI of cells infected with CFSE-labelled *P. gingivalis*. Data from the epithelial cell invasion and antibacterial assays were analysed using Dunnett’s test to determine whether there were differences with and without *Neisseria* spp. The data from the pre-mixed culture system were analysed using Tukey–Kramer’s test to determine whether there were differences between the simultaneous and pre-mixed culture systems. These data are shown as relative mean ± standard deviation with *P. gingivalis* alone as 1.0, and antibacterial assay results are shown as raw colony forming unit values ± standard deviation. A P-value < 0.05 was considered statistically significant.

## RESULTS

### *In-vitro* Evaluation of the Effect of *Neisseria* spp. on *P. gingivalis* Invasion

**Fig 1:**
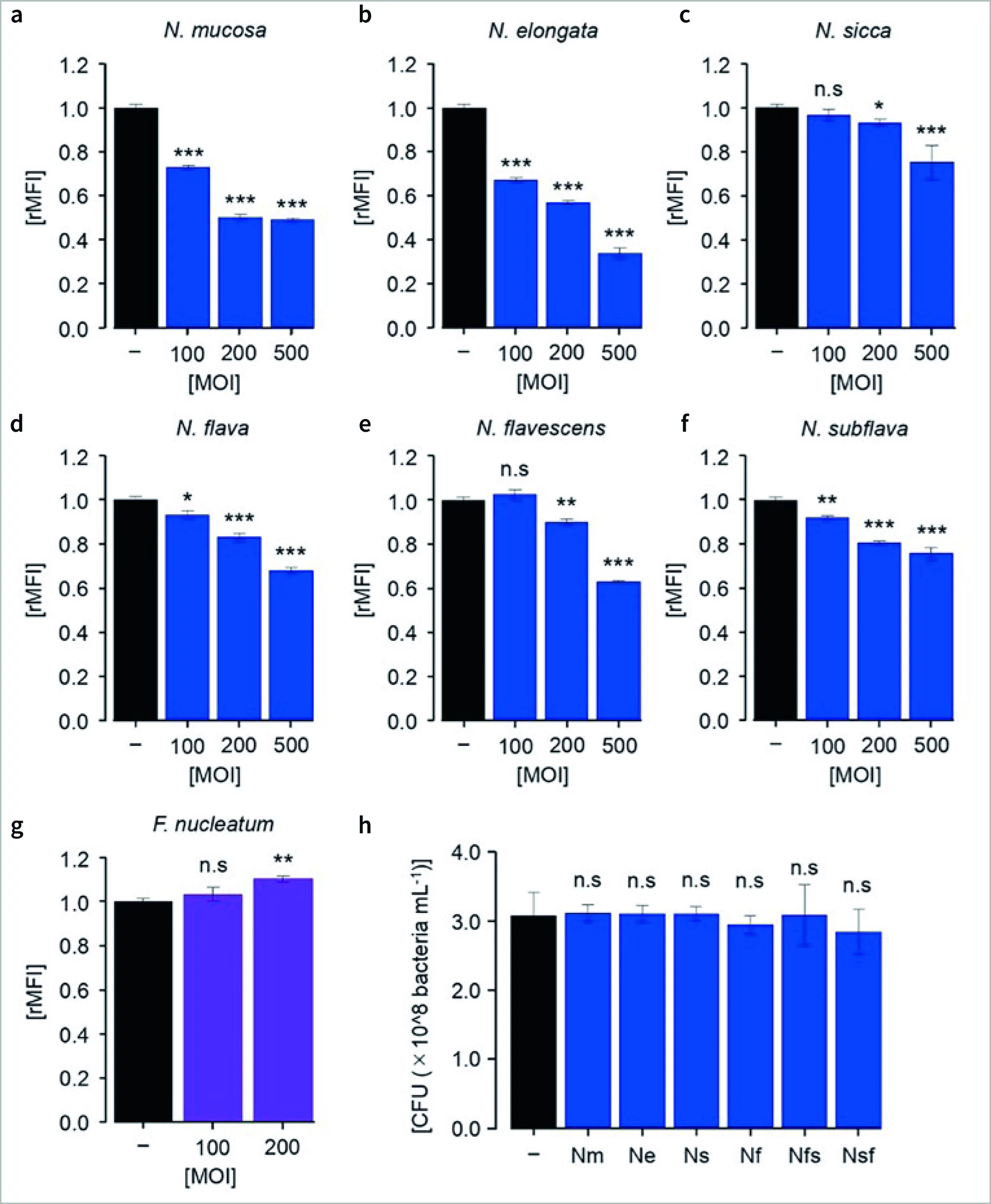
*Neisseria* spp. inhibition of *Porphyromonas gingivalis* invasion. (a) Mono- or co-infection of *P. gingivalis* with *N. mucosa*. (b) Mono- or co-infection of *P. gingivalis* with *N. elongata*. (d) Mono- or co-infection of *P. gingivalis* with *N. sicca*. (d) Mono- or co-infection of *P. gingivalis *with *N. flava*. (e) Mono- or co-infection of *P. gingivalis* with *N. flavescens*. (f) Mono- or co-infection of *P. gingivalis* with *N. subflava*. (g) Mono- or co-infection of *P. gingivalis* with *F. nucleatum*. Relative mean fluorescence intensity [rMFI] is shown as relative mean ± standard deviation with *P. gingivalis* alone as 1.0 (n = 3, n.s = not significant (P > 0.05), *P < 0.05, **P < 0.01, ***P < 0.001, Dunnett’s test). (h) Quantification of the number of viable *P. gingivalis* detected in the antibacterial assay. Colony forming unit (CFU) is shown as relative mean ± standard deviation with *P. gingivalis *alone as 1.0 (n = 3, n.s = not significant (P > 0.05), Dunnett’s test). Nm = *N. mucosa*, Ne = *N. elongata*, Ns = *N. sicca*, Nf = *N. flava*, Nfs = *N. flavescens*, Nsf = *N. subflava*.

To evaluate the effect of *Neisseria* spp. on *P. gingivalis* gingival epithelial cell invasion, we established an *in-vitro* evaluation system based on previous studies.^43^ When CFSE-labelled *P. gingivalis* was co-cultured with *Neisseria* spp. (MOI = 200, 500), all *Neisseria* spp. decreased the fluorescence intensity (Figs 1a–1f), especially for *N. mucosa* and *N. elongata* (Figs 1a and 1b). Conversely, when CFSE-labelled *P. gingivalis* was co-cultured with *F. nucleatum* (MOI = 200), the fluorescence intensity was increased (Fig 1g). The co-culture of CFSE-labelled *P. gingivalis* and *F. nucleatum* (MOI = 500) showed high cytotoxicity at 2 h of infection and did not reach analysable cell numbers (data not shown). Also, we performed an antibacterial assay under anaerobic conditions in the absence of cells, exploiting the inability of *Neisseria* spp. to grow, and evaluated whether *Neisseria* spp. has antibacterial activity against *P. gingivalis* (Fig 1h). No difference was observed in the number of viable *P. gingivalis* colonies between *P. gingivalis* alone and *P. gingivalis* mixed with *Neisseria* spp. (Fig 1h).

### *Neisseria* spp. and *P. gingivalis* Interaction

**Fig 2:**
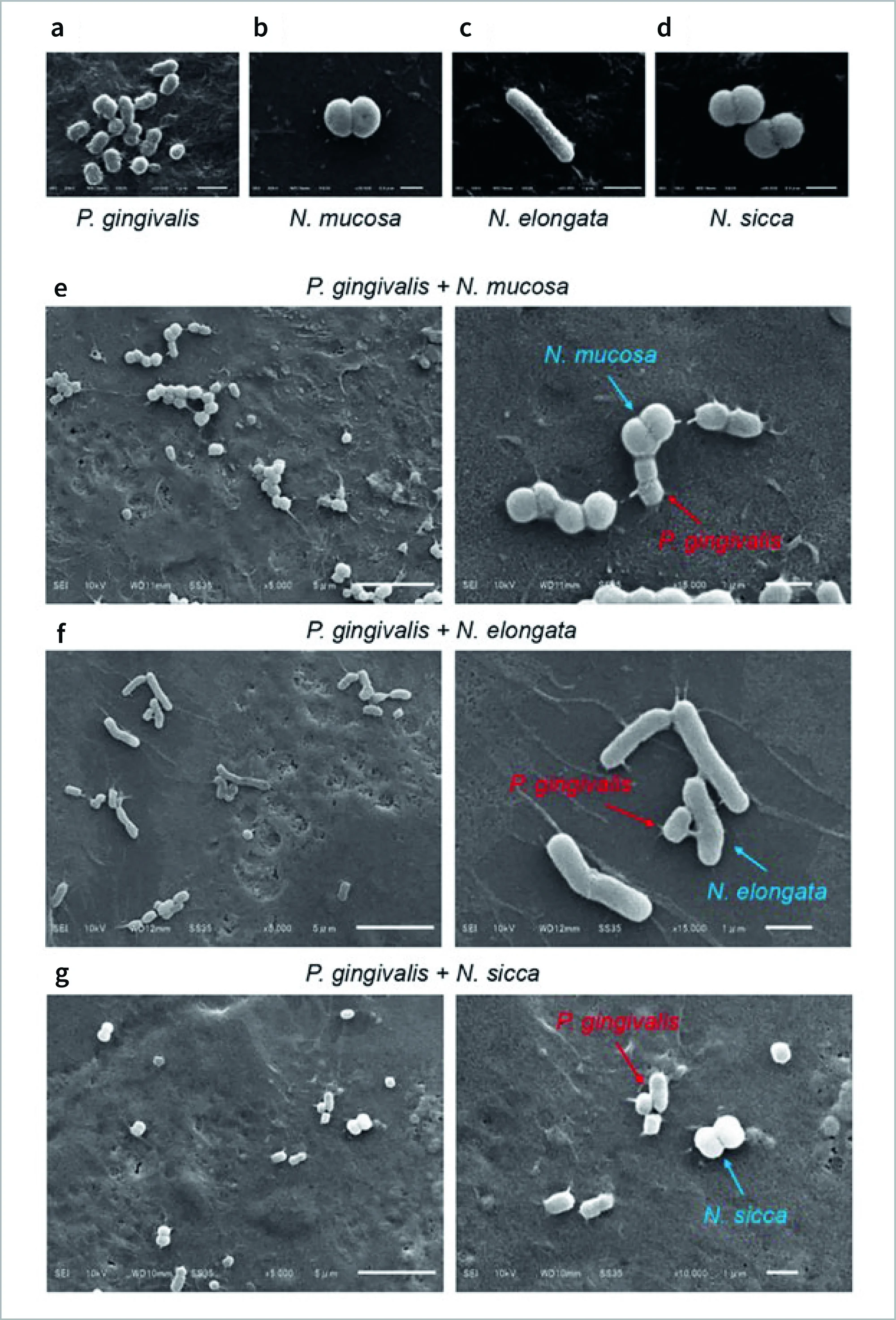
*N. mucosa* and *N. elongata* interacting with *Porphyromonas gingivalis* at a high frequency. (a) Scanning electron microscopy (SEM) image of *P. gingivalis* (scale bar = 1 µm). (b) SEM image of *N. mucosa* (scale bar = 0.5 µm). (c) SEM image of *N. elongata* (scale bar = 1 µm). (d) SEM image of *N. sicca* (scale bar = 0.5 µm). (e) SEM images of *P. gingivalis* and *N. mucosa* co-cultures (left image: scale bar = 5 µm, right image: scale bar = 1 µm). (f) SEM images of *P. gingivalis* and *N. elongata* (left image: scale bar = 5 µm, right image: scale bar = 1 µm). (g) SEM images of *P. gingivalis* and *N. sicca* co-culture (left image: scale bar = 5 µm, right image: scale bar = 1 µm).

To investigate the mechanism of fluorescence intensity suppression by *Neisseria* spp., we used SEM to visualise the interactions between *Neisseria* spp. and *P. gingivalis* on the cell surface. In the analyses, *N. mucosa* and *N. elongata* — which effectively inhibited *P. gingivalis* invasion in the invasion assay — were compared with *N. sicca*, which is known to be present in plaques as well as *N. mucosa* and *N. elongata.* ^13^ For each bacterium, *P. gingivalis* was observed as a coccus or bacillus (Fig 2a), N. mucosa and N. sicca as a diplococcus (Figs 2b and 2d), and *N. elongata* as a bacillus (Fig 2c). *N. mucosa* and *N. elongata*, which showed high inhibition of invasion, interacted with P. gingivalis at most sites (Figs 2e and 2f). In contrast, *N. sicca*, which inhibited *P. gingivalis* invasion less effectively than *N. mucosa* and *N. elongata* did, was located around *P. gingivalis*, but did not interact with *P. gingivalis* in most regions (Fig 2g).

### Effect of Interaction in P. gingivalis Invasion Ability

**Fig 3:**
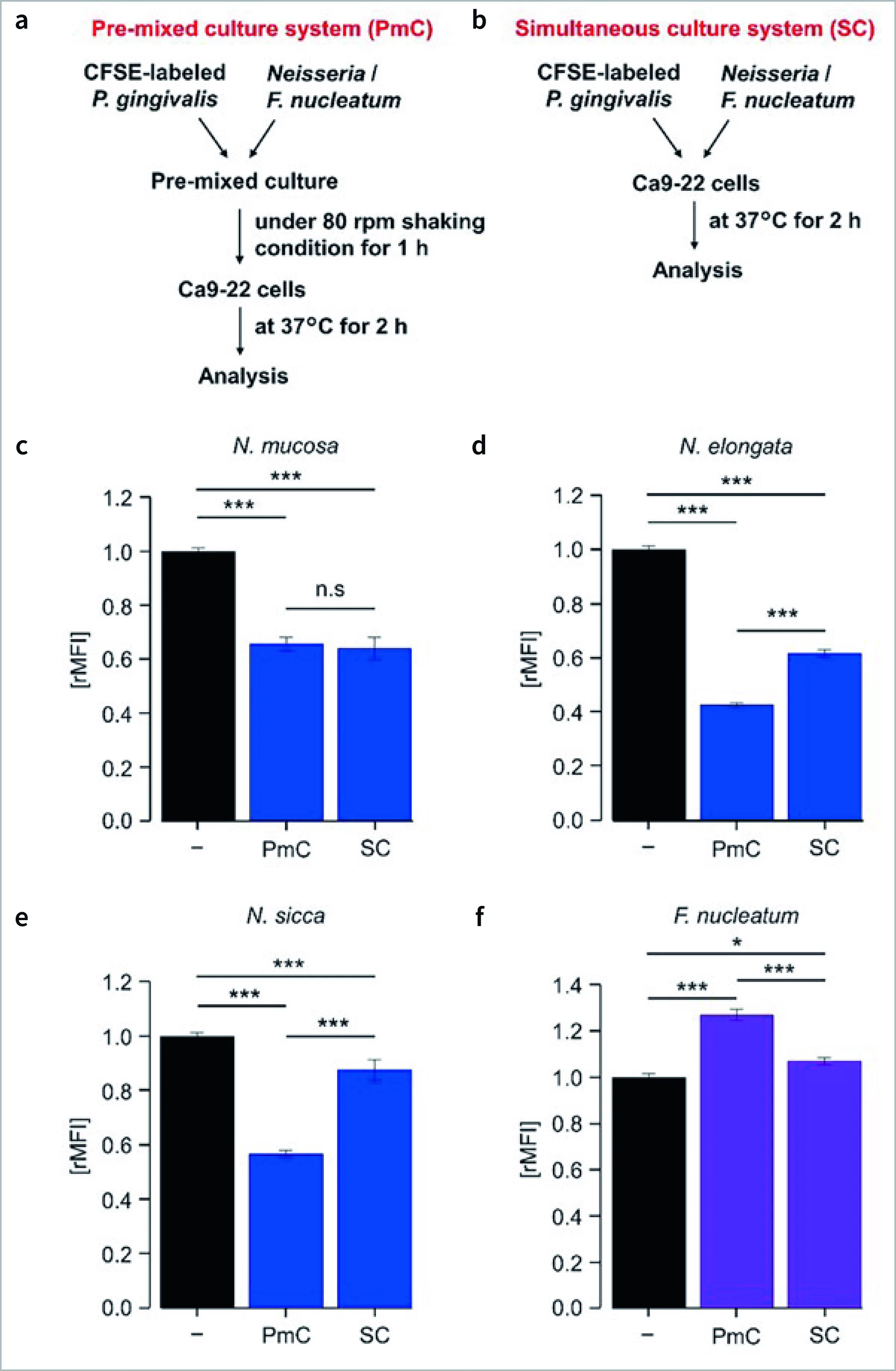
Further inhibitory effect on invasion by *Neisseria* spp. in the pre-mixed culture system. (a, b) Summary of the pre-mixed culture system (PmC) and simultaneous culture system (SC) used for comparison. (c) Mono- or co-infection of *P. gingivalis* with *N. mucosa*. (d) Mono- or co-infection of *P. gingivalis* with *N. elongata*. (e) Mono- or co-infection of *P. gingivalis* with *N. sicca*. (f) Mono- or co-infection of *P. gingivalis* with *F. nucleatum*. Relative mean fluorescence intensity [rMFI] is shown as relative mean ± standard deviation with *P. gingivalis* alone as 1.0 (n = 3–6, n.s = not significant (P > 0.05), *P < 0.05, **P < 0.01, ***P < 0.001, Tukey–Kramer’s test).

To clarify the relationship between fluorescence intensity suppression and bacterial interactions, a pre-mixed culture system was constructed to reproduce the interaction environment (Fig 3a). For comparison, we also tested how the effect changes in the interaction environment using the simultaneous culture system, which was almost the same as that in the invasion assay (Fig 3b). In the pre-mixed culture system, *N. mucosa*, *N. elongata*, and *N. sicca* decreased the fluorescence intensity by 34%, 57%, and 43%, respectively (Figs 3c–3e). Conversely, *F. nucleatum* increased the fluorescence intensity by 26% (Fig 3f). In the simultaneous culture system, *N. mucosa*, *N. elongata*, and *N. sicca* decreased the fluorescence intensity by 35%, 38%, and 13%, respectively (Figs 3c–3e). In contrast, *F. nucleatum* increased the fluorescence intensity by 6% (Fig 3f).

## DISCUSSION

In this study, we aimed to experimentally verify commensal *Neisseria* effects on *P. gingivalis* invasion. The results show that all *Neisseria* spp. inhibited *P. gingivalis* invasion of gingival epithelial cells under conditions where *F. nucleatum* promoted *P. gingivalis* infection as in previous studies (although with a slight increase in this study), and bacterial interaction may be involved. However, the exact mechanism of this interaction with *P. gingivalis* to inhibit invasion remains unclear. The components involved in *P. gingivalis* virulence, such as fimbriae and gingipains are important for infection^14,25,34,53,54^; particularly, fimbriae-related virulence has also been extensively examined using strains with defective fimbriae.^17^ Therefore, further detailed studies, such as utilising *P. gingivalis* mutants lacking fimbriae, are needed to gain a more comprehensive understanding of the interaction. Also, recent studies have shown that *P. gingivalis* and *F. nucleatum* affect periodontitis by inducing epigenetic changes in gingival epithelial cells.^26^ Therefore, it is also necessary to assess the effect of *Neisseria* spp. on these epigenetic changes. In addition, the effects of *P. gingivalis* on *Neisseria* spp. must be considered. Therefore, when assessing bacterial interactions, it is necessary to consider both the effects of virulence factors and epigenetic changes.

Importantly, in the pre-mixed culture system, a further inhibitory effect on invasion was not observed for *N. mucosa*. This suggests that *N. mucosa* inhibits *P. gingivalis* invasion via a different mechanism than that of *N. elongata* and *N. sicca*. *Neisseria* have been shown to have the ability to reduce NO₃^–^ and/or NO₂^–^.^20^ Notably, *N. mucosa* is the only *Neisseria* species that has been reported to possess the ability to reduce not only NO₂^–^ to NO but also NO₃^–^ to NO₂^–^, unlike other *Neisseria* spp., including *N. elongata* and *N. sicca.* ^4^ Whether this ability to reduce NO₃^–^ and/or NO₂^–^ plays a role in the inhibition of invasion is unknown; however, *N. mucosa* may have properties distinct from other *Neisseria* spp. that increase its effectiveness in inhibiting invasion. Therefore, further assessment of the other properties of *N. mucosa* and the mechanism by which it inhibits *P. gingivalis* invasion is required.

In this study, we experimentally demonstrated the beneficial role of *Neisseria* spp. for periodontal disease for the first time. Several studies have reported that *Neisseria* may have beneficial functions for diseases other than periodontal disease. *N. elongata* is more abundant at the supragingival margins,^38^ and *N. subflava* is more abundant on the tongue^55^ in healthy individuals than in individuals with caries. There have also been many reports of low *Neisseria* levels in patients with oral cancer,^10,16,51,56^ and species-level analyses have shown that *N. sicca* and *N. flavescens* are particularly low^3,30,35^ in those cases. Additionally, *Neisseria* spp. has been shown to decrease the proliferation rate of carcinoma cells,^19^ and *N. sicca* plays a role in maintaining genomic stability in the control of oral cancer.^45^ Low levels of *Neisseria*, especially *N. elongata*,^9^ have also been reported in patients infected with influenza A pdm09 virus^27^ and severe acute respiratory syndrome coronavirus 2.^11,12,32^ It has also been confirmed that *Neisseria* is low in patients with oesophageal cancer^28^ and that *N. mucosa* is halved in the oral cavity of patients with inflammatory bowel disease.^42^ These reports suggest that commensal *Neisseria* may be an important oral probiotic against periodontal disease, dental caries, oral cancer, and systemic diseases.

Recent studies have shown that probiotics or synbiotics as new oral care approaches have been proposed as a promising preventative strategy.^39–41,46^ There have been some reports, on the effects of *Lactobacillus* and *Bifidobacterium* on *P. gingivalis*. For example, *Limosilactobacillus fermentum* ALAL020 produces cyclic peptides and shows antibacterial activity,^24^ *Lacticaseibacillus rhamnosus* L8020 inhibits the accumulation of periodontal disease-related pathogens,^33^ and *Bifidobacterium dentium* and *Bifidobacterium longum* specifically reduce the number of viable *P. gingivalis*.^23^ However, there have been few reports on the use of oral commensal bacteria as probiotics. This study evaluated, for the first time, the effects of *Neisseria* spp. on *P. gingivalis* invasion using an *in-vitro* evaluation system and SEM. In addition, since commensal *Neisseria* are significantly increased in the oral cavity by the ingestion of nitrate,^39,47,48^ it is conceivable that this could be a possible approach to prevent periodontal disease through the effects of synbiotics that combine nitrate and beneficial *Neisseria*. In other words, we demonstrated the possibility of using oral *Neisseria* spp. — a commensal bacterium — for the prevention of periodontal disease, unlike conventional probiotics such as *Lactobacillus* and *Bifidobacterium*.

Our study has some limitations. First, the absolute number of *P. gingivalis* that invaded into Ca9-22 cells cannot be estimated based on the fluorescence intensity of our invasion assay. Methods to distinguish adherent bacteria from invading bacteria using antibodies instead of fluorescent labelling have been reported,^22^ and such evaluation should be considered in future research. Second, this study evaluated the effect of *Neisseria* on *P. gingivalis in-vitro*; however, the differences between the present experimental system and real-life clinical conditions are not yet clear. The oral cavity is a complex environment, hosting several bacterial species. Therefore, to verify the effect of *Neisseria* spp. in the oral cavity more reliably and in detail, *in-vivo* and *human* studies are also necessary. Third, we have only evaluated the inhibitory effect of *P. gingivalis* invasion by *Neisseria* spp. on one cell line (Ca9-22 cells) and one *P. gingivalis* strain (type strain: ATCC 33277). Also, the effects of *Neisseria* on *P. gingivalis* were assessed; however, the effects of *P. gingivalis* on *Neisseria* were not assessed. Therefore, it is necessary to understand these interactions in more detail by examining the interactions in multiple cell lines and *P. gingivalis* strains and assessing the effects of *P. gingivalis* on *Neisseria*.

In this study, we found that commensal *Neisseria* inhibited *P. gingivalis* invasion of gingival epithelial cells. To our knowledge, this is the first study to report the effects of *Neisseria* on periodontal pathogens. Furthermore, we found that *N. elongata* and *N. sicca* inhibit *P. gingivalis* invasion through direct interactions. However, the mechanisms by which *N. mucosa* inhibits *P. gingivalis* invasion remain to be elucidated and require further analysis. This study suggests that *Neisseria*, as a probiotic or synbiotic commensal bacteria in the oral cavity, may represent a new approach for preventing periodontal disease.

### Acknowledgements

This research was financially supported by the Kao Corporation. The authors would like to thank Taichi Konno (Processing Development Research, Kao Corporation) for technical assistance with the SEM experiments and Editage (http://www.editage.com) for editing and reviewing this manuscript for enhanced English language.

## References

[ref1] Andrian E, Grenier D, Rouabhia M (2004). In vitro models of tissue penetration and destruction by Porphyromonas gingivalis. Infect Immun.

[ref2] Avila M, Ojcius DM, Yilmaz O (2009). The oral microbiota: living with a permanent guest. DNA Cell Biol.

[ref3] Baraniya D, Jain V, Lucarelli R, Tam V, Vanderveer L, Puri S (2020). Screening of health-associated oral bacteria for anticancer properties in vitro. Front Cell Infect Microbiol.

[ref4] Barth KR, Isabella VM, Clark VL (2009). Biochemical and genomic analysis of the denitrification pathway within the genus Neisseria. Microbiology (Reading).

[ref6] Belstrom D, Holmstrup P, Damgaard C, Borch TS, Skjodt MO, Bendtzen K (2011). The atherogenic bacterium Porphyromonas gingivalis evades circulating phagocytes by adhering to erythrocytes. Infect Immun.

[ref7] Bui FQ, Almeida-da-Silva CLC, Huynh B, Trinh A, Liu J, Woodward J (2019). Association between periodontal pathogens and systemic disease. Biomed J.

[ref8] Cai Z, Lin S, Hu S, Zhao L (2021). Structure and function of oral microbial community in periodontitis based on integrated data. Front Cell Infect Microbiol.

[ref9] Callahan N, Hattar M, Barbour T, Adami GR, Kawar N (2022). Oral microbial taxa associated with risk for SARS-CoV-2 infection. Front Oral Health.

[ref10] Chen X, Winckler B, Lu M, Cheng H, Yuan Z, Yang Y (2015). Oral microbiota and risk for esophageal squamous cell carcinoma in a high-risk area of China. PLoS One.

[ref13] Donati C, Zolfo M, Albanese D, Tin Truong D, Asnicar F, Iebba V (2016). Uncovering oral Neisseria tropism and persistence using metagenomic sequencing. Nat Microbiol.

[ref14] Frias-Lopez J, Duran-Pinedo A (2012). Effect of periodontal pathogens on the metatranscriptome of a healthy multispecies biofilm model. J Bacteriol.

[ref15] Geng F, Liu J, Guo Y, Li C, Wang H, Wang H (2017). Persistent Exposure to porphyromonas gingivalis promotes proliferative and invasion capabilities, and tumorigenic properties of human immortalized oral epithelial cells. Front Cell Infect Microbiol.

[ref16] Guerrero-Preston R, Godoy-Vitorino F, Jedlicka A, Rodriguez-Hilario A, Gonzalez H, Bondy J (2016). 16S rRNA amplicon sequencing identifies microbiota associated with oral cancer, human papilloma virus infection and surgical treatment. Oncotarget.

[ref17] Hasegawa Y, Nagano K (2021). Porphyromonas gingivalis FimA and Mfa1 fimbriae: current insights on localization, function, biogenesis, and genotype. Jpn Dent Sci Rev.

[ref18] How KY, Song KP, Chan KG (2016). Porphyromonas gingivalis: an overview of periodontopathic pathogen below the gum line. Front Microbiol.

[ref19] Hu X, Shen X, Tian J (2021). The effects of periodontitis associated microbiota on the development of oral squamous cell carcinoma. Biochem Biophys Res Commun.

[ref20] Hyde ER, Andrade F, Vaksman Z, Parthasarathy K, Jiang H, Parthasarathy DK (2014). Metagenomic analysis of nitrate-reducing bacteria in the oral cavity: implications for nitric oxide homeostasis. PLoS One.

[ref21] Ikeda E, Shiba T, Ikeda Y, Suda W, Nakasato A, Takeuchi Y (2020). Japanese subgingival microbiota in health vs disease and their roles in predicted functions associated with periodontitis. Odontology.

[ref22] Inaba H, Nomura R, Kato Y, Takeuchi H, Amano A, Asai F (2019). Adhesion and invasion of gingival epithelial cells by Porphyromonas gulae. PLoS One.

[ref23] Jasberg H, Soderling E, Endo A, Beighton D, Haukioja A (2016). Bifidobacteria inhibit the growth of Porphyromonas gingivalis but not of Streptococcus mutans in an in vitro biofilm model. Eur J Oral Sci.

[ref24] Kawai T, Ohshima T, Tanaka T, Ikawa S, Tani A, Inazumi N (2022). Limosilactobacillus (Lactobacillus) fermentum ALAL020, a probiotic candidate bacterium, produces a cyclic dipeptide that suppresses the periodontal pathogens Porphyromonas gingivalis and Prevotella intermedia. Front Cell Infect Microbiol.

[ref25] Kuboniwa M, Lamont RJ (2010). Subgingival biofilm formation. Periodontol 2000.

[ref26] Larsson L, Kavanagh NM, Nguyen TVN, Castilho RM, Berglundh T, Giannobile WV (2022). Influence of epigenetics on periodontitis and peri-implantitis pathogenesis. Periodontol 2000.

[ref27] Leung RK, Zhou JW, Guan W, Li SK, Yang ZF, Tsui SK (2013). Modulation of potential respiratory pathogens by pH1N1 viral infection. Clin Microbiol Infect.

[ref28] Li H, Luo Z, Zhang H, Huang N, Li D, Luo C (2021). Characteristics of oral microbiota in patients with esophageal cancer in China. Biomed Res Int.

[ref29] Liu G, Tang CM, Exley RM (2015). Non-pathogenic Neisseria: members of an abundant, multi-habitat, diverse genus. Microbiology (Reading).

[ref30] Liu Y, Li Z, Qi Y, Wen X, Zhang L (2022). Metagenomic analysis reveals a changing microbiome associated with the depth of invasion of oral squamous cell carcinoma. Front Microbiol.

[ref31] Mao S, Park Y, Hasegawa Y, Tribble GD, James CE, Handfield M (2007). Intrinsic apoptotic pathways of gingival epithelial cells modulated by Porphyromonas gingivalis. Cell Microbiol.

[ref32] Merenstein C, Liang G, Whiteside SA, Cobian-Guemes AG, Merlino MS, Taylor LJ (2021). Signatures of COVID-19 severity and immune response in the respiratory tract microbiome. mBio.

[ref33] Oda Y, Kawano R, Murakami J, Kado I, Okada Y, Nikawa H (2023). Effect of Lacticaseibacillus rhamnosus L8020 on the abundance of periodontal pathogens in individuals with intellectual disability: a randomized clinical trial. Quintessence Int.

[ref34] Ogawa T, Yagi T (2010). Bioactive mechanism of Porphyromonas gingivalis lipid A. Periodontol 2000.

[ref35] Peters BA, Wu J, Pei Z, Yang L, Purdue MP, Freedman ND (2017). Oral microbiome composition reflects prospective risk for esophageal cancers. Cancer Res.

[ref36] Poole S, Singhrao SK, Chukkapalli S, Rivera M, Velsko I, Kesavalu L (2015). Active invasion of Porphyromonas gingivalis and infection-induced complement activation in ApoE-/- mice brains. J Alzheimers Dis.

[ref38] Qudeimat MA, Alyahya A, Karched M, Behbehani J, Salako NO (2021). Dental plaque microbiota profiles of children with caries-free and caries-active dentition. J Dent.

[ref39] Rosier BT, Buetas E, Moya-Gonzalvez EM, Artacho A, Mira A (2020). Nitrate as a potential prebiotic for the oral microbiome. Sci Rep.

[ref40] Rosier BT, Marsh PD, Mira A (2018). Resilience of the oral microbiota in health: mechanisms that prevent dysbiosis. J Dent Res.

[ref41] Rosier BT, Moya-Gonzalvez EM, Corell-Escuin P, Mira A (2020). Isolation and characterization of nitrate-reducing bacteria as potential probiotics for oral and systemic health. Front Microbiol.

[ref42] Said HS, Suda W, Nakagome S, Chinen H, Oshima K, Kim S (2014). Dysbiosis of salivary microbiota in inflammatory bowel disease and its association with oral immunological biomarkers. DNA Res.

[ref43] Saito A, Inagaki S, Kimizuka R, Okuda K, Hosaka Y, Nakagawa T (2008). Fusobacterium nucleatum enhances invasion of human gingival epithelial and aortic endothelial cells by Porphyromonas gingivalis. FEMS Immunol Med Microbiol.

[ref44] Sedghi L, DiMassa V, Harrington A, Lynch SV, Kapila YL (2021). The oral microbiome: role of key organisms and complex networks in oral health and disease. Periodontol 2000.

[ref45] Shen X, Zhang B, Hu X, Li J, Wu M, Yan C (2022). Neisseria sicca and Corynebacterium matruchotii inhibited oral squamous cell carcinomas by regulating genome stability. Bioengineered.

[ref46] Teughels W, Newman MG, Coucke W, Haffajee AD, Van Der Mei HC, Haake SK (2007). Guiding periodontal pocket recolonization: a proof of concept. J Dent Res.

[ref47] Vanhatalo A, Blackwell JR, L’Heureux JE, Williams DW, Smith A, van der Giezen M (2018). Nitrate-responsive oral microbiome modulates nitric oxide homeostasis and blood pressure in humans. Free Radic Biol Med.

[ref48] Velmurugan S, Gan JM, Rathod KS, Khambata RS, Ghosh SM, Hartley A (2016). Dietary nitrate improves vascular function in patients with hypercholesterolemia: a randomized, double-blind, placebo-controlled study. Am J Clin Nutr.

[ref49] Wirth R, Maroti G, Liptak L, Mester M, Al Ayoubi A, Pap B (2022). Microbiomes in supragingival biofilms and saliva of adolescents with gingivitis and gingival health. Oral Dis.

[ref50] Yamashita Y, Takeshita T (2017). The oral microbiome and human health. J Oral Sci.

[ref52] Yilmaz O, Verbeke P, Lamont RJ, Ojcius DM (2006). Intercellular spreading of Porphyromonas gingivalis infection in primary gingival epithelial cells. Infect Immun.

[ref53] Yoshimura F, Murakami Y, Nishikawa K, Hasegawa Y, Kawaminami S (2009). Surface components of Porphyromonas gingivalis. J Periodontal Res.

[ref54] Zenobia C, Hajishengallis G (2015). Porphyromonas gingivalis virulence factors involved in subversion of leukocytes and microbial dysbiosis. Virulence.

[ref55] Zhang D, Takeshita T, Furuta M, Kageyama S, Asakawa M, Nambu K (2021). Tongue microbiota composition and dental caries experience in primary school children. mSphere.

[ref56] Zhao Q, Yang T, Yan Y, Zhang Y, Li Z, Wang Y (2020). Alterations of oral microbiota in chinese patients with esophageal cancer. Front Cell Infect Microbiol.

